# Any Way the Wind Blows Does Really Matter in Lichen Response to Air Pollution from an Oil Refinery

**DOI:** 10.3390/toxics13030160

**Published:** 2025-02-25

**Authors:** Maja Maslać Mikulec, Saša Likić, Oleg Antonić, Mirta Tkalec

**Affiliations:** 1 Department of Biology, Faculty of Science, University of Zagreb, 10 000 Zagreb, Croatia; saslikic@yahoo.com; 2 Geonatura Ltd., 10 000 Zagreb, Croatia; oantonic@geonatura.hr; 3 Biosistemi Grupa Ltd., 10 000 Zagreb, Croatia; 4 Department of Biology, Josip Juraj Strossmayer University of Osijek, 31 000 Osijek, Croatia

**Keywords:** air quality, biomonitoring, bioindicators, generalized linear models (GLMs), lichen vitality, bioaccumulation, *Flavoparmelia caperata*

## Abstract

Lichens serve as effective bioindicators for air pollution studies, yet most biomonitoring research focuses primarily on the distance from pollution sources, often neglecting wind data that could elucidate the spread of airborne pollutants. In our previous study in Slavonski Brod, Croatia, we utilized data from a monitoring station, emphasizing the impact of meteorological conditions, particularly wind, on the dispersal of pollutants from a neighbouring oil refinery. To gain a deeper understanding of air pollution dynamics, here, we studied lichen vitality—measured through photochemical efficiency and photosynthetic pigments—alongside the metal (Ni, Zn, Cd, Pb) and non-metal (sulphur and nitrogen) content in native lichen species *Flavoparmelia caperata* across 17 plots within a 20 km radius of the refinery. Our analysis employed generalized linear models (GLMs) to incorporate various environmental predictors, including distance from the refinery, direction-specific wind speed and frequency, vegetation density, and the orientation of lichen samples with respect to north and the refinery. Findings show that pollution levels are significantly influenced, not only by distance but also by direction-specific wind patterns, underscoring the necessity of including these variables in future biomonitoring studies and highlighting a critical need for air quality management interventions.

## 1. Introduction

Air pollution is one of the most pressing challenges of our time, significantly affecting public and individual health, harming the environment, and contributing to climate change [1]. Among various monitoring methods, biomonitoring is particularly valuable as it provides evidence of the cumulative effects that airborne pollutants exert on organisms. This approach reflects a time-integrated outcome influenced by both air quality and local climatic conditions [2]. Furthermore, biomonitoring allows for greater sampling density compared to standard monitoring stations, enabling the identification of specific sources of high-risk areas [3]. There are limitations in using organisms for air quality monitoring, such as natural variability in a heterogeneous environment, with some other factors inducing disturbance [4]. Nevertheless, lichens have been successfully used as biomonitors and bioindicators.

Lichens, recognized as reliable bioindicators, are frequently assessed for physiological changes (vitality) driven by air pollution, including alterations in photochemical efficiency and chlorophyll integrity [5]. The chlorophyll *a* fluorescence parameters are widely used to assess the photosynthetic performance of lichens, which is affected by various stress conditions, including air pollution [6,7,8,9,10]. Likewise, the ratios of photosynthetic pigments shift due to air pollution, with reductions seen in chlorophyll *a* (Chl *a*) value and phaeophytinization quotient (ratio of Chl *a* to phaeophytin *a*) [6,9,11,12,13].

Lichens have the ability to accumulate airborne pollutants, including heavy metals, reflecting the environmental profile from which they are sampled [14,15,16]. Research indicates that lichens can also capture non-metallic compounds, such as sulphur [17] and nitrogen—an even bigger environmental concern lately [18]. The atmospheric deposition of sulphur and nitrogen compounds not only adversely impacts organisms (including lichens) directly as these are pollutants, but it also leads to acidification, which may enhance the mobilization of toxic metals. Additionally, nitrogen compounds contribute to eutrophication [19].

Consequently, lichens have been effectively used in studies to pinpoint and characterize pollution from various sources, including oil refineries [20,21]. These sites are significant contributors to air pollution through the release of numerous pollutants, including particulate matter (PM), which contains heavy metals (e.g., arsenic, molybdenum, nickel, selenium, vanadium, lead), along with other pollutants such as sulphur oxides, methane, carbon dioxide, nitrous oxide, persistent organic pollutants (POPs), and volatile organic compounds (VOCs) [22].

In our previous study in the Slavonski Brod area of Croatia [23], where we used data from a monitoring station, we identified the nearby oil refinery as a major source of air pollution, primarily contributing to SO_2_ and H_2_S emissions, while also significantly affecting NO_2_ and PM levels. This study confirmed that wind direction and speed are critical predictors of air pollution. However, the incorporation of wind data is frequently overlooked in current lichen biomonitoring research, or it is limited in scope. Existing biomonitoring studies, involving lichens, that include wind data often only provide qualitative analyses of wind patterns (e.g., [24]), highlighting a significant gap in understanding the full impact of wind on pollutant dynamics and lichen vitality. For instance, Cristofolini et al. [25] investigated potential environmental predictors related to air pollution and lichen biodiversity in the Italian Prealps, but did not incorporate wind data or analyze lichen physiological changes or element concentrations. Additionally, although other environmental predictors, such as precipitation data and topography, have been studied in relation to lichen species distribution (e.g., [26]), the influence of wind data remains largely unexplored.

Thus, the objectives of this research are to (1) assess the impact of the oil refinery on lichen vitality and the content of metal and non-metal pollutants, thereby demonstrating the efficacy of lichens as bioindicators and biomonitors; (2) identify key environmental predictors, including meteorological factors (wind data), that could further elucidate the dynamics of air pollution in the area while monitoring changes in lichen physiology and pollutant accumulation; and (3) evaluate the benefits and limitations of this biomonitoring approach, offering recommendations for future studies to enhance the integration of environmental data in understanding the effects of air pollution on organisms.

## 2. Materials and Methods

### 2.1. Study Area

Slavonski Brod, home to approximately 50,000 residents [27], is recognized as the most polluted city in Croatia, primarily due to emissions from the nearby oil refinery located across the Sava River, which serves as the natural state border with Bosnia and Herzegovina. Reports from the Meteorological and Hydrological Service (DHMZ) show that the two monitoring stations within the city have recorded alarming contents of several pollutants, including sulphur dioxide (SO_2_), hydrogen sulphide (H_2_S), ozone (O_3_), and fine particles (PM_2.5_), since the refinery resumed operations in 2008 after an 18-year hiatus [28]. Although nitrogen dioxide (NO_2_) levels exceed permissible limits at one of the monitoring stations, the duration of these exceedances has not been sufficiently alarming compared to the other pollutants. According to our previous study [23], maximum concentrations for PM_2.5_ (~60 μg m^−3^), NO_2_ (~20 μg m^−3^), SO_2_ (~18 μg m^−3^), and H_2_S (~3 μg m^−3^) are typically observed during the colder months. In contrast, O_3_ concentrations reach their peak levels (~70 μg m^−3^) during periods of increased photolytic activity, which occurs predominantly at midday in the summer months.

### 2.2. Sampling Design

**Figure 1:**
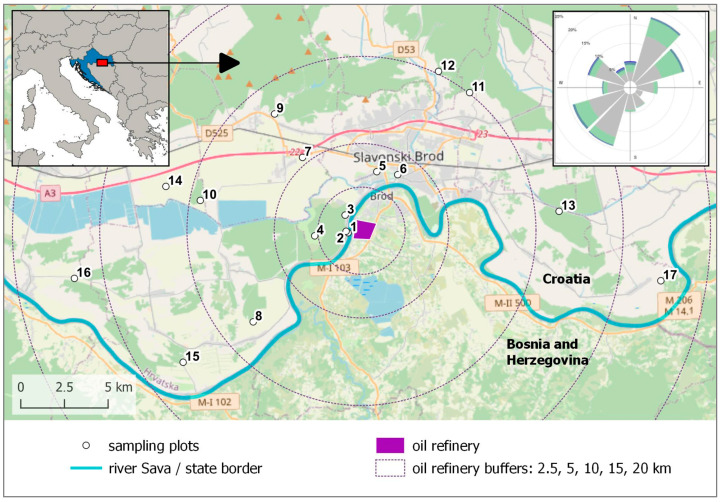
The area with locations of the sampling plots, the city of Slavonski Brod (Croatia), and the oil refinery in Bosnia and Herzegovina across the state border (Sava River). Sampling point 5 is in the location of the standard monitoring station. The wind rose (upper right image) for Slavonski Brod during the period from 2011 to 2014 is taken from Jeričević et al. [23]. Background image © OpenStreetMap contributors, CC BY-SA.

Slavonski Brod is located on a plain terrain along the Sava River where agricultural land dominates outside the city area, but with patches of oak forests, whose bark is a suitable substrate for the lichen species used in this study. We chose to sample *Flavoparmelia caperata* (L.) Hale, a species abundant in this forest type at the required trunk height, which is commonly used in biomonitoring studies with native lichens [29,30]. According to Nimis [31], this species occurs in natural or semi-natural habitats up to moderately disturbed areas (agricultural areas, small settlements, etc.); in places with no eutrophication up to weak eutrophication; and on acid substrata, such as non-eutrophicated bark of *Quercus*, up to subacid to subneutral substrata (e.g., on bark of *Sambucus*). Permission for the collection of lichens was given by the Ministry of Environmental and Nature Protection. To assess possible biological effects caused by the airborne pollutants from the refinery, 17 sampling plots were chosen within a 20 km radius, with a higher density of sampling plots closer to the refinery, where higher impact was expected due to proximity to the pollution source. Thus, in the 0–2.5 km radius, there were three sampling plots, three in the 2.5–5 km radius, four in the 5–10 km radius, five in the 10–15 km radius, and two in the 15–20 km radius. Sampling plots were located at the edges of lowland oak forests to ensure that all lichens collected experienced similar microhabitat conditions, meaning they were growing on the same type of bark and were exposed to comparable levels of precipitation and pH. Since the refinery is in the territory of Bosnia and Herzegovina, the samples were only collected on the Croatian side of the border (Figure 1). Sampling plot 5 was located at the site of the monitoring station, from which we obtained data for our previous research [23].

A minimum of 10 samples were collected in December 2015 at a height of 1–3 m from up to 10 trees from each sampling plot (the maximum size of a plot was a 30 m radius). All sampling was carried out in a five-day period. Additional parameters for each sample were also collected, namely, the orientation of the lichen on the tree (with respect to north and respect to refinery), and the vegetation density around the sampling point. Samples were stored in paper bags and kept in a dry place at room temperature (20 to 25 °C) until chlorophyll fluorescence analysis was performed within ten days of sampling. In the most recent literature [30], sample washing and the use of whole thalli were abandoned in favour of manual debris cleaning and the use of peripheral portions of thalli. This is what we carried out before the rest of the analysis. After cleaning, samples were kept in a cold (+4 °C) and dry place until the remaining analyses were completed (within two months’ time).

### 2.3. Laboratory Analysis

#### 2.3.1. Lichen Vitality

In order to perform chlorophyll fluorescence analysis, lichens were placed on filter paper in a Petri dish, dampened with distilled water, and acclimated in laboratory conditions (light intensity of 60 µmol photons m^−2^ s^−1^ at 22 ± 2 °C) overnight. Chlorophyll fluorescence measurements were performed on lichen thalli adapted to darkness for 30 min using light-withholding clips. Measurements were carried out with a FluorPen FP100 (Photon Systems Instruments, Brno, Czech Republic). Modulated light of weak intensity was applied to measure the minimal level of fluorescence (F_0_), followed by a short flash of saturating light to assess the maximal level of fluorescence (F*_m_*). The minimal/maximal level of fluorescence in the light-adapted state and the level of steady-state fluorescence (F*s*) were measured by means of saturating pulses (>3000 μmol m^−2^ s^−1^), applied on top of the actinic light (100 μmol m^−2^ s^−1^). The maximum photochemical quantum efficiency of photosystem II (F*_v_*/F*_m_*), nonphotochemical quenching (NPQ), and the coefficient of photochemical quenching (Q*p*) were calculated according to Maxwell and Johnson [32]. Additionally, the chlorophyll fluorescence decrease ratio (RFd), which correlates with the potential CO_2_ fixation, was determined [33].

Photosynthetic pigments were extracted from powdered dry lichen thalli (10–15 mg) with 1.5 mL dimethyl sulfoxide, with the addition of CaCO_3_. Samples were incubated for 40 min in a warm (65 °C) ultrasonic bath and then centrifuged for 10 min at room temperature and 10,000× *g*. The supernatants were separated and used for spectrophotometric measurements. The absorbance of the extracts was measured using a UV–VIS spectrophotometer (Specord 40, Analytik Jena AG, Jena, Germany). The quantification of chlorophylls and carotenoids, as well as chlorophyll integrity, was estimated in accordance with the previously described procedures [13,34].

For chlorophyll fluorescence and photosynthetic pigment content, as indicators of lichen vitality, at least nine replicates were measured for each sampling plot. In total, 14 variables were obtained: F*_v_*/F*_m_*, Q*p*, NPQ, RFd, chlorophyll *a* (Chl *a*), chlorophyll *b* (Chl *b*), total chlorophyll (TChl), phaeophytinization quotient (PQ*a*), total carotenoid (TCar) content, and chlorophyll *a* and *b* ratio (Chl *a*/Chl *b*).

#### 2.3.2. Metal and Non-Metal Content Analysis

The metal content (lead—Pb, zinc—Zn, cadmium—Cd, nickel—Ni) in microwave-digested dry lichen thalli (~100 mg) was analyzed using an ELAN DRC-e (Perkin Elmer, Waltham, MA, USA) inductively coupled plasma mass spectrometer (ICP-MS) [35]. Powdered dry lichen thalli (~25 mg) were also analyzed for total nitrogen (N) and sulphur (S) content via the dry combustion method using an elemental analyser Vario Macro Cube (Elementar, Langenselbold, Germany). At least three replicates were measured for each sampling plot. Recoveries and reproducibility were checked by the analysis of procedural blanks and calibration standards. The precision of analysis was estimated by the coefficient of variation in three replicates and was found to be within 5%.

### 2.4. Data Analysis

All geospatial analysis was performed in software package QGIS 3.16.11. All statistical analysis was performed in software package Statistica 14.0.

#### 2.4.1. Basic Statistics

A non-parametric Kruskal–Wallis test was used to detect statistically significant differences between groups of samples collected on different plots for each of the 17 dependent variables (lichen vitality: F*_v_*/F*_m_*, Q*p*, NPQ, RFd, Chl *a*, Chl *b*, TChl, PQ*a*, Chl *a*/Chl *b*, TCar; non-metals: N, S; metals: Ni, Zn, Cd, Pb).

#### 2.4.2. Using Bioaccumulation Scale for Metals

For metal contents, we employed a standardized methodology for native lichen surveys [30] to create maps by dividing the mean values by review-based background element concentration values (BEC; µg g⁻^1^ dw), for the lichen species *Flavoparmelia caperata*, utilizing the standard bioaccumulation scale (refer to Tables S1 and S2).

#### 2.4.3. Building GLMs

Eight variables of lichen vitality (F*_v_*/F*_m_*, NPQ, Q*p*, RFd, TChl, PQ*a*, TCar, Chl *a*/Chl *b*) were input in the principal component analysis (PCA), aiming to reduce the number of dependent variables of lichen vitality. This was performed together with Varimax normalized rotation of those components with eigenvalues greater than one [36]. Variables of lichen vitality were reduced via four principal components (see Table S2, Supplementary material) which explained 85.8% of total variability, where the first component (F1; 36.2% of total variance) represents all pigment variables (TChl, PQ*a*, TCar, Chl *a*/Chl *b*), the second (F2; 24.8%) represents NPQ and RFd, the third (F3; 12.9%) represents Q*p,* and the fourth (F4; 11.8%) represents F*_v_*/F*_m_*. All the close correlations in this PCA were positive.

At the next step, the missing data of dependent variables (non-metal and metal concentrations, and four principal components of lichen vitality) were filled with the respective variable average for each sampling plot. Before building the regression models, we performed univariate statistical analysis (correlation matrix) to observe particular relationships between non-metal and metal content, and four principal components of lichen vitality.

For the last step, independent variables were explored as environment predictors of lichen vitality and emission levels (N, S, Ni, Zn, Cd, Pb) within the surroundings of the refinery, which were collected at plot, tree, and sample levels. Thus, the independent variables collected for each plot included (1) the distance of the plot from the oil refinery, (2) the annual wind frequency from the direction of the oil refinery, and (3) respective annual mean wind speed. Independent variables collected for each tree only included (4) the estimation of vegetation density around the sampled tree. Finally, independent variables collected for each sample included (5) the relative orientation of the lichen on the tree trunk with respect to the north and (6) the relative orientation of the lichen on the tree trunk with respect to the oil refinery direction. Details on sampling procedures, calculations, and range values of all variables used in data analysis (dependent and independent) are presented in the Appendix A (Table A1).

In order to explain the spatial variability of dependent variables, two groups of linear models were built (for each dependent variable): for Model A only the distance from the oil refinery was used as an independent variable (simple linear regression), and for Model B all the independent variables described were used (generalized linear model; GLM). In the second approach, each of the ten dependent variables (four principal components, as well as non-metal and metal contents) were fitted by all GLMs, assembled in terms of all possible combinations of independent variables (including linear and bivariate interaction terms). The model with an optimal subset of independent variables (for the respective dependent variable) was selected using Mallows’ Cp statistics [37] and was then used for the final interpretation of spatial patterns of lichen physiological response and bioaccumulation due to air pollution in the vicinity of the oil refinery.

## 3. Results

In this study, 17 plots were established for lichen sampling; however, not a single species of lichen was found on any of the oak trees in the two plots closest to the refinery (<2 km), indicating a “lichen desert”, or an area with extremely high levels of air pollution.

### 3.1. Basic Statistics

For the remaining 15 sampling plots, the results of the Kruskal–Wallis tests indicate the existence of statistically significant differences (at least at *p* = 0.05) among the plots across all dependent variables (Table 1). Although none of the dependent variables exhibited a clear correlation with distance from the oil refinery, some potential patterns did emerge (Table 1, Tables S4 and S5). For instance, almost all measured variables displayed either their highest (F*_v_*/F*_m_*, pigment variables, N, S, Zn, Pb) or lowest (Q*p*, NPQ, RFd) values in plots 5 and/or 6, while plot 4 exhibited the opposite trend for these variables. This observation underscores the necessity of a model that incorporates parameters beyond mere distance from the pollution source.

### 3.2. Bioaccumulation Classifications for Metals

**Figure 2:**
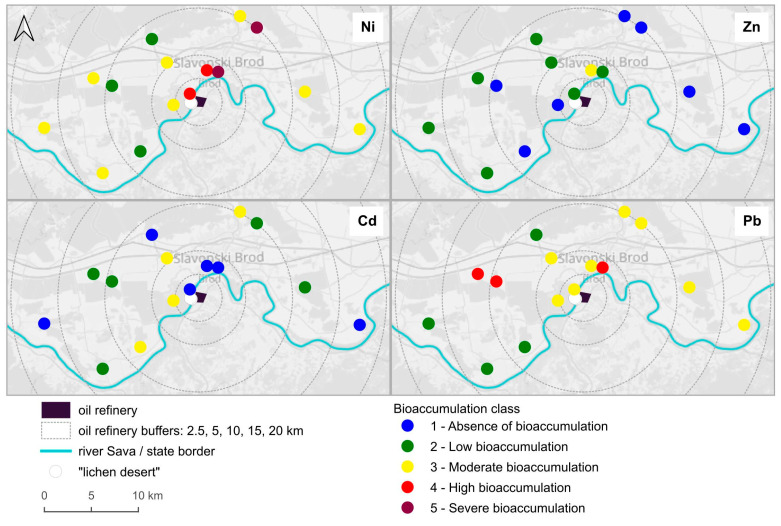
Bioaccumulation classes for Ni, Zn, Cd, and Pb in *Flavoparmelia caperata* on sampling plots (classified according to Cecconi et al. [30]). On the two plots closest to the refinery no lichens were found (“lichen desert”). Background image © OpenStreetMap contributors, CC BY-SA.

According to the bioaccumulation classification [30], severe accumulation was found for Ni, Pb exhibited high bioaccumulation levels, while Cd and Zn showed moderate bioaccumulation (Figure 2). It is again evident that none of the measured metal contents exhibited a clear correlation with distance from the oil refinery, but different patterns emerged—severe and high bioaccumulation of Ni in the northeast direction, up to 10 km from the refinery, and high bioaccumulation of Pb on two plots about 10 km away from the refinery, but in a different direction. We can also observe that at sampling plot 5, located at the standard monitoring station, Zn is the only metal that shows the highest level of bioaccumulation, while the other heavy metals have their highest concentrations at different sampling plots.

### 3.3. Correlation Matrix

After the principal component analysis (PCA) reduced the number of dependent variables associated with lichen vitality, a correlation matrix was generated. This matrix indicated the existence of positive correlations among N, S, Ni, Zn, and F1 (Table 2). Cd displayed negative correlations with N, S, Zn, and F1. Factor F1, which encompasses all pigment variables, exhibited positive correlations with both non-metals and various metals, consistent with findings in Table 1, where these variables demonstrated the highest concentrations across the same plots. Conversely, F2, representing NPQ and RFd, showed a negative correlation with S and a positive correlation with Ni. Factor F3, representing Q*p*, was negatively correlated with N, S, Zn, and Pb. Lastly, F4, denoting F*_v_*/F*_m_*, demonstrated a positive correlation, solely with N. The four principal components related to lichen vitality did not exhibit correlations with one another.

### 3.4. Model Results

**Figure 3:**
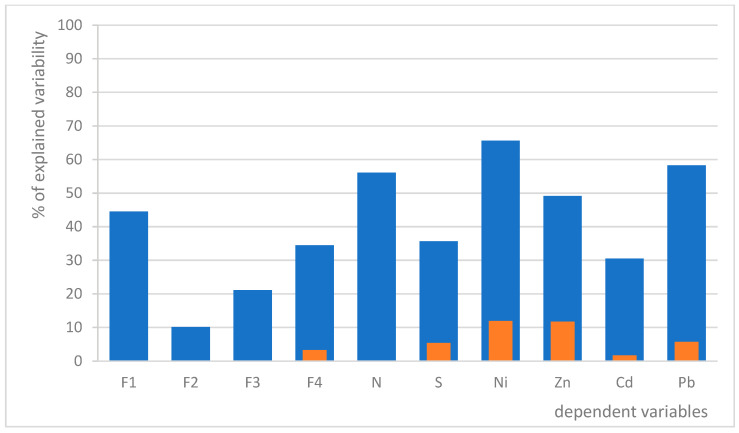
A comparison of the predictive power of two GLMs, one with distance as the only environmental predictor (Model A; orange) and the other with a combination of 6 environmental predictors (Model B; blue). Only significant relations are shown.

We developed two generalized linear models (GLMs) to assess the impact of various predictors on lichen vitality. Model A utilizes distance from the refinery as the sole linear predictor, while Model B incorporates an optimized linear combination of six environmental parameters (Table 3), both models for each dependent variable. Our results indicate that distance alone accounts for significantly less variability in each dependent variable compared to the model that includes the selected environmental parameters (Figure 3). This suggests that the additional environmental factors play a substantial role in influencing the concentrations of non-metals and metals, as well as overall lichen vitality.

Analyzing environmental parameters as independent linear terms in GLMs (without considering interactions terms), the distance from the refinery was, expectedly, negatively correlated with most variables in both models. The only positive correlations were in model A for F3 and F4, and in model B for Cd. Wind frequency had a positive correlation with S, F1, F2 and Ni, and a negative correlation with F4 and Pb, while wind speed had a negative correlation with F3 and Ni, and a positive one with Zn and Pb. The orientation of the lichen on the trunk relative to the refinery had a negative correlation with F3 (like for distance and wind speed) and positive with Zn and Cd. Vegetation density only had a negative correlation with Pb. The orientation of the lichen on the trunk relative to north, when taken as an independent estimator, did not have an impact on any variables.

Among six environmental parameters included in Model B, the distance from the refinery accounted for the second highest number (i.e., 22) of significant contributions to the explanation of the total variability. Direction-specific wind speed and frequency were also notable predictors, with 24 and 19 significant contributions, respectively, while for the orientation of the lichen on the trunk relative to the refinery and to true north, along with vegetation density, 12, 2 and 15 significant contributions were recorded.

## 4. Discussion

This study represents the first biomonitoring effort in Slavonski Brod, where we identified the strong effect of air pollution on lichens, especially near the oil refinery, which has been identified as a major source of pollutants based on existing monitoring station data analysis [23]. We demonstrated that the use of lichens as biomonitors and bioindicators provides valuable insights that complement data obtained from standard monitoring stations. Lichen responses revealed spatial variability in pollutant distribution, highlighting areas of higher pollution that are not adequately represented by the monitoring station. Furthermore, this approach yielded additional information on air pollution of the area, which will be detailed in the following sections.

### 4.1. “Lichen Desert”

Alarmingly, our findings indicated the existence of a “lichen desert” within two plots closest to the refinery (<2 km), where no lichens were found on the trees. This phenomenon typically correlates with high level of SO_2_ which, along with nitrogen oxides, is the main cause of toxic acidification [38]. Hawksworth and Rose [39] developed a scale describing changes in epiphytic lichen communities in relation to SO_2_ concentrations, which has been shown to be robust in subsequent studies [40]. This scale indicates that lichens cannot survive in environments where SO_2_ concentrations exceed 150 µg m^−3^, while a sensitive species such as *F. caperata* disappears with SO_2_ levels higher than 50 µg m^−3^. This suggests that the SO_2_ levels in the areas closest to the oil refinery in our study are likely above this threshold. Indeed, Jeričević et al. [23] reported that monitoring stations recorded high hourly SO_2_ concentrations ranging from 300 to 820 µg m^−3^, particularly during stable atmospheric boundary layer (SABL) conditions, which promote the accumulation of pollutants. While improved regulations and reduced pollutant concentrations have led to the recolonization of “lichen deserts” in many urban and industrial areas [41,42], our study indicates the continued severe impact of air pollution in this region. Here, we could argue that other pollutants coming from the refinery, such as heavy metals or even hydrocarbons, are expected to cumulatively contribute to the “lichen desert”, along with SO_2_. It is known that acid–moisture depositions containing heavy metals can significantly reduce lichen survival. For example, in the lichen *Bryoria fuscescens,* the critical concentration of Ni was >7 µg g⁻^1^ in the presence of acidity and >20 µg g⁻^1^ in absence of acidity [43].

### 4.2. Lichen Vitality and Bioaccumulation

For most of dependent variables, the highest values were obtained for plots 5 and 6, which are 5 km away from the oil refinery. But, unlike similarly distant plot 4, they are in the prevailing wind direction coming from the oil refinery (Figure 1). Furthermore, plots 5 and 6 are situated in or around the city, where nitrogen oxides should play a significant role in at least some of pollutants’ levels. On these plots, the nitrogen content exceeded 20 mg g⁻^1^ dw, comparable to the nitrogen levels found in *F. caperata* sampled at a nitrogen-rich pig stock farm [44], where the values ranged from 20.6 to 25.6 mg g⁻^1^ dry weight. Ochoa-Hueso and Manrique [45] found that even a modest increase in nitrogen availability, commonly found in urban and agricultural environments, can lead to higher chlorophyll *a* content in lichen *Cladonia foliacea* without necessarily improving photosynthetic activity. In contrast, excessively high nitrogen concentrations have been linked to reductions in both pigment content and chlorophyll fluorescence parameters, especially in sensitive epiphytic species, and *F. caperata* is considered N-sensitive [8,46,47]. Our findings reveal the existence of a positive correlation between nitrogen levels and both pigment content (F1) and photosynthetic efficiency, measured as F*_v_*/F*_m_* (F4), indicating that increased nitrogen levels may drive lichens to adjust their metabolism to enhance pigment synthesis, thereby maintaining lower nitrogen levels to avoid negative impacts on photosynthesis and pigment metabolism. This phenomenon has been documented across various lichen species and experimental settings, with some researchers referring to it as the “fertilizing effect” [48]. Considering that this study investigates native lichens that have thrived in this environment for years, it is plausible that they have adapted their metabolic processes to manage higher nitrogen levels. However, the intricate relationship between nitrogen—an essential but potentially detrimental factor—and lichen viability poses interpretative challenges, particularly since nitrogen significantly influences lichen photobionts in terms of chlorophyll production and the synthesis of photosystem proteins [6]. Also, it is possible that the negative impact of high N concentrations is reduced by the availability of other essential mineral nutrients [6].

The positive correlations between accumulated N, S, Ni, and Zn could potentially be explained by them having same source of pollution, with the highest levels again being seen on the plots that are in the dominant wind direction from the refinery, rather than the closest ones, indicating the influence of other environmental factors, as further discussed in the next section. The sulphur content found in our samples is slightly higher than previously reported in lichen *Ramalina celastri* near industry (up to 1.17 mg g⁻^1^) [49], probably because it is a different lichen species. Sulphur concentrations in lichens are expected to correlate with sulphur pollution from the oil refineries [21], which is significant in this case, as already mentioned. Photosynthetic performance is in general reduced by sulphur [50], which is in agreement with our results that show the existence of a negative correlation between sulphur and F2 and F3, chlorophyll fluorescence parameters which reflect photosynthetic activity. However, F3 was negatively correlated with Pb and Zn content, and so it is also possible that these heavy metals contributed to the reduction in photosynthetic activity. Certain metals are known to reduce the efficiency of the photosynthetic apparatus [51], as well as to reduce the concentrations of photosynthetic pigments [52], although this effect depends on the lichen species. For example, increased contents of Zn, Cd, Cu, and Ni in *Diploschistes muscorum* and of Zn and Ni in *Cladonia rei* decreased the contents of photosynthetic pigments, whereas concentrations of Pb had a positive effect in all lichen species [52]. We found a positive correlation between Ni, Zn, and Pb and pigment variables (F1). On the contrary, the Pb content was negatively related to the Chl *a* content, the Chl *a*/*b* ratio, and the PQ value in *F. caperata* from the urban area of Kolkata in India [53]. We can speculate that our model explains those samples, which are in the part of the range of effects where nitrogen induces pigment synthesis, and where elevated sulphur and heavy metals are not damaging for the pigment metabolism. Moreover, it has been found that lichens growing naturally at highly polluted sites have higher pigment contents as an adaptation to air pollution [54].

The bioaccumulation classification for metals was developed by Cecconi et al. [30], incorporating species considerations derived from numerous pollution studies in Italy, including those focused on oil refineries. Oil refineries are recognized as significant sources of heavy metal pollution, particularly for elements such as arsenic (As), molybdenum (Mb), Ni, selenium (Se), vanadium (V), and Pb [22], with Ni and V being considered as tracers of oil-burning and/or oil-refining industries [55]. Since the phase-out of leaded gasoline significantly reduced emissions (Croatia in 2006, Bosnia and Hercegovina in 2010), today, the primary sources of Pb in air pollution are the processing of metals and ore, leaded aviation gasoline, and emissions from battery manufacturing, coal burning, typecasting, and older structures, with the highest Pb levels found near lead smelters [56]. Nevertheless, it has been shown that historical gasoline-derived Pb remains an important source of Pb due to its persistence and effective remobilization [57]. In our study, we documented the severe bioaccumulation of Ni and high levels of Pb in *F. caperata* samples. However, these levels were not detected at sampling plot 5, which also serves as the site of the monitoring station. Notably, during the year prior to this research, measurements of Ni, Cd, and Pb in PM_10_ began on the monitoring station, with regulatory standards defining a limit for the yearly average of these metals (20 ng m^−3^, 5 ng m^−3^, 0.5 µg m^−3^), which were officially reported as not being exceeded, categorizing the air quality as “good” for these pollutants in 2015 [58]. However, a closer examination of the data reveals that the annual limit was exceeded on numerous occasions throughout the year for Ni; it was merely the average that remained below the threshold (Figure S1). In this context, lichens provide a more accurate representation of the impact of pollution on organisms, enabling us to critically reevaluate the validity of annual average limit values. This is particularly relevant in regions like Slavonski Brod, where seasonal variations can significantly influence pollution dynamics.

### 4.3. Wind Matters

The GLM revealed important correlations between various independent variables and lichen responses, showcasing the intricate dynamics of environmental factors and their impact on lichen vitality and bioaccumulation. Notably, the distance from the refinery consistently showed a negative correlation with most lichen vitality and bioaccumulation variables across both models. This trend aligns with expectations; as the distance from the pollution source increases, the potential for exposure to harmful pollutants diminishes, leading to higher lichen vitality. The positive correlations observed in model A for F3 (indicating Q*p*) and F4 (indicating F*_v_*/F*_m_*) are also to be expected, as these values are expected to be lower when the stress is higher [6,7,8,9,10]. In model B, there is a positive correlation for Cd, which indicates a possibility that some other source of Cd might be in the area.

Wind frequency emerged as a key variable with notable correlations. It positively correlated with S, F1 (which reflects pigment content), F2 (indicating NPQ), and Ni. This suggests that increased wind activity may enhance the deposition and dispersal of pollutants, contributing to higher levels of certain elements in the lichen tissues and impact lichen vitality. Conversely, there is a negative correlation between wind frequency and F4 (indicating F*_v_*/F*_m_*) and Pb. These results for F4 align with expectations, showing lower values under stress conditions [6,7,8,9,10], in this case caused by more frequent winds from the main pollution source. On the other hand, the negative correlation with Pb suggests that another source may be contributing to the elevated Pb concentrations in the study area, as illustrated in Figure 2. Similarly, wind speed demonstrated negative correlations with F3 (indicating Q*p*) and Ni, indicating a potential negative impact on photosynthetic efficiency and lichen vitality, while showing positive correlations with Zn and Pb. This duality reinforces the idea that wind speed influences the dynamics of pollutant accumulation, possibly affecting how lichens interact with their environment.

The orientation of lichens on the trunk relative to the refinery exhibited a negative correlation with F3, mirroring the effects of distance and wind speed. It also showed positive correlations with Zn and Cd, suggesting that lichens oriented towards the refinery may be more exposed to certain contaminants. Interestingly, when considering the orientation of lichen relative to the north as an independent estimator, we found no significant impact on the variables measured. Vegetation density around the sampled trees had a singular negative correlation with Pb levels, indicating that denser vegetation may reduce the accumulation of lead in lichens. This highlights the potential protective role of surrounding plant life in mitigating pollutant exposure, at least for Pb.

The existence of significant interaction terms in GLMs (products of the particular independent variables) suggests that abovementioned relationships are even more complex, including possible synergistic or antagonistic mutual effects among particular environmental variables that additionally influence lichen vitality and/or the bioaccumulation level.

In our study, we are pioneering the quantitative integration of wind data into a model focused on lichen studies. While other environmental factors, such as precipitation and topography, have been explored as predictors of lichen species distribution (e.g., [26]), wind data have been largely overlooked. Previous ecophysiological studies on lichens performed in the field typically only considered the distance from major pollution sources as an independent variable (e.g., [34,59,60]). This is surprising, since air pollution studies that do not use biological models like lichens have shown that wind direction and speed are important predictors of air pollution (e.g., [61]).

Our findings indicate that distance alone accounts for significantly less variability in each dependent variable compared to our second model, which incorporates selected environmental parameters, most notably direction-specific wind speed and frequency. These recorded correlations align logically with pollutant dispersion patterns and their impacts on lichen viability, suggesting that variations in wind dynamics can significantly influence the distribution of pollutants and subsequently the concentrations of both non-metals and metals, as well as overall lichen vitality. Still, the distance from the refinery was negatively correlated with most variables, in both models, which was expected [7,8,9,62].

Previous biomonitoring studies that address wind often only provide qualitative insights into wind patterns (e.g., [24]). On the other hand, Cristofolini et al. [25] investigated environmental predictors related to air pollution and lichen biodiversity in the Italian Prealps but did not incorporate wind data. Also, they were focused on biodiversity and did not study the effect of air pollution on lichen physiological changes (lichen vitality) or elemental concentrations (bioaccumulation). By quantitatively including wind data, our research fills a critical gap and advances the understanding of environmental influences on lichen distributions and health.

It is important to note that while our models account for a substantial portion of the data variability, they only explain part of the overall picture, as expected in a complex environment. In this context, it is crucial to acknowledge that many pollutants in the area are also generated by sources such as traffic and wood heating, further contributing to the intricacies of pollutant dynamics and their effects on lichen vitality. In this area, there is a mixture of pollutants. Therefore, we can even expect a hormesis-based cross-phenomenon [63], which has not yet been addressed in lichens.

## 5. Conclusions

While it is widely recognized that wind influences the dispersion of air pollutants, our study is the first to quantitatively integrate wind data into a model focusing on lichens, which are well regarded as biomonitors and bioindicators. Our findings demonstrate that wind speed and frequency account for significantly more variability in lichen responses than distance from pollution sources alone. The air pollution challenges in Slavonski Brod stem not only from the refinery’s proximity but also from its positioning relative to the city, with prevailing winds blowing pollutants directly toward urban areas. This connection is evident in the elevated levels of several measured variables found at sampling locations within and around the city. Notably, the data indicate that, where present, lichens have adapted to the pollution.

As the first biomonitoring effort in Slavonski Brod—known as Croatia’s most significant air pollution hotspot due to its proximity to an oil refinery—this study reinforces the refinery’s role as a major environmental concern. It highlights lichens as effective bioindicators, revealing an alarming “lichen desert” phenomenon, where air pollution levels are so high that lichens cannot grow. Additionally, the bioaccumulation data showed concerning levels of nickel and lead that exceeded expectations based on traditional air quality metrics and legal limits. These results collectively underscore the significant air pollution problem present in the area, since such high levels of pollutants must have a significant impact on human health and the environment.

Despite the inherent limitations of using biological organisms for air quality monitoring—such as natural variability in heterogeneous environments—lichens have proven to be reliable indicators of pollution for decades. Some countries, like Italy and the USA, have successfully integrated lichens into their air quality monitoring frameworks, a practice still lacking in many regions, including Croatia. We hope this research will promote the adoption of lichens in air quality monitoring strategies and policies. Going forward, we recommend that future biomonitoring studies assess wind data alongside the distance from pollution sources.

## Figures and Tables

**Table 1:** Measured values of dependent variables in sampled native *Flavoparmelia caperata* lichens per plot (mean; coloured by variable in a colour scale—highest number is the darkest blue colour, white is the lowest): chlorophyll fluorescence parameters (F*_v_*/F*_m_*—maximum photochemical quantum efficiency of photosystem II, NPQ—nonphotochemical quenching, Q*p*—coefficient of photochemical quenching, RFd—fluorescence decrease ratio); pigment variables in mg g⁻^1^ dw (Chl *a*—chlorophyll *a*, Chl *b*—chlorophyll *b*, TChl—total chlorophyll, TCar—total carotenoids) and no measuring unit (PQ*a*—phaeophytinization quotient); non-metal (S—sulphur, N—nitrogen) content in mg g⁻^1^; metal (Ni—nickel, Zn—zinc, Cd—cadmium, Pb—lead) content in µg dw. Plots are lined up from the closest to the farthest from the refinery. On the plots closest to the refinery (1 and 2), no lichens were found. The mean ± standard deviation is presented in Table S4. Other basic statistical values are shown in Table S5.

Plot	F*_v_*/F*_m_*	Q*p*	NPQ	RFd	Chl *a*	Chl *b*	TChl	PQ*a*	Chl *a*/Chl *b*	TCar	N	S	Ni	Zn	Cd	Pb
3	0.71	0.28	0.82	0.76	1.09	0.43	1.52	0.84	2.47	0.4	14.62	1.66	6.12	50.6	0.12	5.15
4	0.67	0.26	0.92	0.84	0.62	0.29	0.9	0.73	2.15	0.31	8.44	1.35	3.61	34.43	0.4	5.34
5	0.69	0.19	0.61	0.52	1.74	0.66	2.40	0.75	2.60	0.62	21.04	2.01	4.82	80.50	0.17	7.34
6	0.74	0.22	0.71	0.64	2.41	0.86	3.27	0.94	2.81	0.49	20.92	1.8	6.28	55.6	0.15	11.13
7	0.68	0.26	0.83	0.7	1.16	0.44	1.6	0.84	2.64	0.36	15.81	1.53	3.54	38.37	0.5	5.48
8	0.73	0.27	0.64	0.6	1.1	0.46	1.56	0.79	2.41	0.41	13.97	1.65	2.54	30.83	0.5	2.61
9	0.7	0.27	0.71	0.61	1.56	0.55	2.11	0.97	2.81	0.44	17.98	1.64	2.4	40	0.12	3.41
10	0.67	0.23	0.87	0.8	0.78	0.34	1.12	0.77	2.32	0.3	9.5	1.31	1.97	25.47	0.25	8.62
11	0.7	0.24	0.91	0.82	1.64	0.59	2.23	0.91	2.79	0.49	17.6	1.63	9.21	30.1	0.28	5.05
12	0.72	0.2	0.85	0.76	0.88	0.41	1.29	0.83	2.29	0.29	10.79	1.39	3.8	27.37	0.43	5.91
13	0.65	0.22	0.78	0.72	0.63	0.3	0.93	0.8	2.09	0.28	9.19	1.34	3.63	19.37	0.32	5.68
14	0.73	0.22	0.71	0.65	1.1	0.42	1.52	0.84	2.52	0.35	14.67	1.52	3.06	43	0.22	8.62
15	0.73	0.21	0.66	0.63	1.06	0.42	1.48	0.76	2.49	0.38	17.31	1.56	3.2	53.3	0.23	4.04
16	0.71	0.26	0.84	0.76	1.13	0.43	1.56	0.85	2.6	0.42	15.97	1.57	2.81	48.4	0.11	3.65
17	0.72	0.28	0.63	0.58	1.19	0.45	1.64	0.82	2.64	0.42	13.86	1.63	2.85	25.5	0.18	5.79
H	66.45	41.78	25.63	27.07	82.03	75.49	82.29	61.08	53.22	71.16	35.84	27.31	25.21	30.92	32.09	31.19
*p*(H)	0.0000	0.0001	0.0288	0.0189	0.0000	0.0000	0.0000	0.0000	0.0000	0.0000	0.0011	0.0175	0.0326	0.0057	0.0039	0.0052
N	149	149	149	149	136	136	136	136	136	136	59	59	45	45	45	45

H and *p*-values computed by a Kruskal–Wallis one-way analysis of variance on ranks; N—sample size.

**Table 2:** Pearson’s correlation coefficients among N, S, Ni, Zn, Cd, Pb and factors of lichen vitality. Bold denotes *p* < 0.05.

	N	S	Ni	Zn	Cd	Pb	F1	F2	F3	F4
N	1.000									
S	**0.795**	1.000								
Ni	**0.303**	**0.281**	1.000							
Zn	**0.650**	**0.611**	**0.145**	1.000						
Cd	**−0.310**	**−0.190**	−0.023	**−0.280**	1.000					
Pb	0.100	0.099	**0.223**	**0.180**	−0.121	1.000				
F1	**0.645**	**0.518**	**0.354**	**0.344**	**−0.328**	**0.158**	1.000			
F2	−0.130	**−0.171**	**0.172**	−0.131	0.060	0.050	0.000	1.000		
F3	**−0.203**	**−0.180**	−0.094	**−0.327**	0.069	**−0.304**	0.000	0.000	1.000	
F4	**0.165**	0.030	−0.089	0.041	−0.100	−0.047	0.000	0.000	0.000	1.000

N—nitrogen; S—sulphur; Ni—nickel; Zn—zinc; Cd—cadmium; Pb—lead content; F1, F2, F3, F4—four principal components of lichen vitality.

**Table 3:** Results of optimized generalized linear models (GLMs) explaining lichen vitality factors (see Table S1) and non-metal (S, N) and metal (Ni, Zn, Cd, Pb) content as a function of various environmental predictors: Model A—the only parameter is the distance between the refinery and the plot; Model B—six environmental parameters were used. The parameters (or combination of parameters) which did not have a significant effect on any of the variables are written in italics. Significant relations are shown in bold.

MODEL		F1	F2	F3	F4	N	S	Ni	Zn	Cd	Pb
	R^2^	0.01	0.00	0.01	**0.03**	0.01	**0.05**	**0.12**	**0.12**	**0.02**	**0.06**
A	F	2.17	1.09	3.32	**7.53**	2.44	**11.93**	**26.89**	**26.38**	**4.26**	**12.55**
	*p* (F)	0.14	0.30	0.07	**0.01**	0.12	**0.00**	**0.00**	**0.00**	**0.04**	**0.00**
	(intercept)	**+**	**+**	**−**	**−**	**+**	**+**	**+**	**+**	**+**	**+**
	d_ref	**−**	**−**	**+**	**+**	**−**	**−**	**−**	**−**	**−**	**−**
	R^2^	**0.45**	**0.10**	**0.21**	**0.34**	**0.56**	**0.36**	**0.66**	**0.49**	**0.31**	**0.58**
B	F	**26.57**	**8.21**	**9.52**	**17.76**	**41.62**	**18.64**	**61.81**	**31.72**	**15.01**	**45.40**
	*p* (F)	**0.00**	**0.00**	**0.00**	**0.00**	**0.00**	**0.00**	**0.00**	**0.00**	**0.00**	**0.00**
	(intercept)	+	−	+	+	+	+	+	+	−	+
	d_ref	−	n.s.	−	n.s.	−	−	−	n.s.	+	n.s.
	v_den	n.s.	n.s.	n.s.	n.s.	n.s.	n.s.	n.s.	n.s.	n.s.	−
	a_ref	n.s.	n.s.	−	n.s.	n.s.	n.s.	n.s.	+	+	n.s.
	*a_north*	n.s.	n.s.	n.s.	n.s.	n.s.	n.s.	n.s.	n.s.	n.s.	n.s.
	w_frq	+	+	n.s.	−	n.s.	+	+	n.s.	n.s.	−
	w_spd	n.s.	n.s.	−	n.s.	n.s.	n.s.	−	+	n.s.	+
	d_ref*v_den	+	n.s.	n.s.	n.s.	+	+	n.s.	+	−	+
	d_ref*a_ref	n.s.	n.s.	n.s.	+	−	n.s.	n.s.	n.s.	n.s.	n.s.
	v_den*a_ref	n.s.	n.s.	n.s.	−	n.s.	n.s.	n.s.	n.s.	n.s.	n.s.
	d_ref*a_north	n.s.	n.s.	n.s.	n.s.	n.s.	n.s.	n.s.	n.s.	n.s.	n.s.
	v_den*a_north	n.s.	n.s.	n.s.	n.s.	n.s.	n.s.	n.s.	n.s.	n.s.	n.s.
	a_ref* a_north	n.s.	n.s.	n.s.	−	n.s.	n.s.	n.s.	n.s.	n.s.	n.s.
	d_ref*w_frq	n.s.	n.s.	−	n.s.	+	n.s.	n.s.	+	n.s.	+
	v_den* w_frq	n.s.	n.s.	n.s.	+	n.s.	n.s.	−	n.s.	n.s.	n.s.
	a_ref*w_frq	n.s.	−	n.s.	n.s.	n.s.	n.s.	n.s.	n.s.	n.s.	n.s.
	a_north*w_frq	n.s.	n.s.	n.s.	n.s.	n.s.	n.s.	n.s.	n.s.	n.s.	n.s.
	d_ref*w_spd	n.s.	n.s.	+	n.s.	n.s.	n.s.	+	−	n.s.	−
	v_den* w_spd	−	n.s.	n.s.	−	−	−	n.s.	n.s.	+	n.s.
	a_ref*w_spd	+	n.s.	n.s.	n.s.	+	+	n.s.	n.s.	−	n.s.
	a_north*w_spd	n.s.	n.s.	n.s.	n.s.	n.s.	n.s.	n.s.	n.s.	+	n.s.
	w_frq*w_spd	−	−	+	n.s.	n.s.	−	−	−	n.s.	n.s.

Independent variables used in analysis as estimators of spatial distribution of pollution from refinery (including quadratic and bivariate interaction terms): (1) the distance of sampling plot from the oil refinery (d_ref), (2) the annual wind frequency coming from the oil refinery’s direction (w_frq), (3) the respective annual mean wind speed (w_spd), (4) the estimation of vegetation density around the sampled tree (v_den), (5) the relative orientation of the lichen on the tree with respect to the north (a_north), (6) the relative orientation of the lichen on the tree with respect to the oil refinery direction (a_ref). N—nitrogen content; S—sulphur content; F1, F2, F3, F4—the four principal components of lichen vitality; R^2^—the coefficient of determination; F—the proportion of explained and unexplained variability (F—statistics); *p* (F)—the probability of F—statistics; n.s.—non-significant; plus (+) denotes a significantly positive correlation; minus (−) denotes a significantly negative correlation. Only the independent variables which were significant estimators (at a probability level of *p* = 0.05) for at least one dependent variable are presented.

## Data Availability

The raw data presented in this study are available on request from the corresponding author.

## Supplementary Materials

The following supporting information can be downloaded at: https://www.mdpi.com/article/10.3390/toxics13030160/s1, Table S1. Correlations of measured variables of lichen vitality with the first four principal components (factor loadings); Table S2. Review-based BECs (µg g⁻^1^ dw) for the epiphytic lichen species *Flavoparmelia caperata* [30]; Table S3. Bioaccumulation classes for native lichens used in this study (adapted from Cecconi et al. [30]); Table S4. Measured values of dependent variables in sampled native lichens per plot (mean ± standard deviation); Table S5. Measured values of dependent variables in sampled native lichens per plot (median; range); Figure S1. Trends in daily average nickel (Ni) concentrations (ng mg^−3^) in the PM_10_ fraction at the Slavonski Brod-1 monitoring station in 2015.

## Abbreviations

The following abbreviations are used in this manuscript:

GLM	generalized linear model
BEC	Background element concentration
dw	Dry weight
SO_2_	Sulphur dioxide
H_2_S	Hydrogen sulphide
O_3_	Ozone
PM_2.5_	Particulate matter (diameter ≤ 2.5 micrometres)
NO_2_	Nitrogen dioxide
Ni	Nickel
Zn	Zinc
Cd	Cadmium
Pb	Lead
N	Nitrogen
S	Sulphur
Chl *a*	Chlorophyll *a*
Chl *b*	Chlorophyll *b*
TChl	Total chlorophyll
PQ*a*	Phaeophytinization quotient
TCar	Total carotenoids
F_*v*_/F_*m*_	Maximum photochemical quantum efficiency of photosystem II
NPQ	Nonphotochemical quenching
Q*p*	Coefficient of photochemical quenching
RFd	Chlorophyll fluorescence decrease ratio
OR	Oil refinery
DHMZ	Meteorological and Hydrological Service (Croatia)
PCA	Principal component analysis
SABL	Stable atmospheric boundary layer

## Appendix A

**Table A1:** Dependent variables (a–j) and environmental parameters (1–6) used in this study.

No.	Variable	Abbreviation	Unit	Range/ Categories	Comments
a	Nitrogen (N) content	N	mg g⁻^1^ dw	5.96–28.81	N concentration was measured in 59 individuals
b	Sulphur (S) content	S	mg g⁻^1^ dw	1.20–2.27	S concentration was measured in 59 individuals
c	Nickel (Ni) content	Ni	µg g⁻^1^ dw	1.74–14.1	Ni concentration was measured in 45 individuals
d	Zinc (Zn) content	Zn	µg g⁻^1^ dw	14.9–94.7	Zn concentration was measured in 45 individuals
e	Cadmium (Cd) content	Cd	µg g⁻^1^ dw	0.09–1.22	Cd concentration was measured in 45 individuals
f	Lead (Pb) content	Pb	µg g⁻^1^ dw	2.08–15.7	Pb concentration was measured in 45 individuals
g	1st principal component of lichen vitality	F1	unitless	−2.30–3.09	Factor representing pigment variables (see Table S2)
h	2nd principal component of lichen vitality	F2	unitless	−1.71–3.94	Factor representing chlorophyll fluorescence variables—NPQ and RFd (see Table S2)
i	3rd principal component of lichen vitality	F3	unitless	−2.34–2.49	Factor representing chlorophyll fluorescence variable—Q*p* (see Table S2)
j	4th principal component of lichen vitality	F4	unitless	−3.13–2.04	Factor representing chlorophyll fluorescence variable—F*_v_*/F*_m_* (see Table S2)
1	Distance from the OR	d_ref	m	1273–17,358	Calculated on plot level
2	Frequency of the wind coming from OR direction	w_frq	%	0.61–12.68	Calculated on plot level from DHMZ data (2010–2015) *
3	Average wind speed from OR direction	w_spd	m s⁻^1^	0.8–1.61	Calculated on plot level from DHMZ data (2010–2015) *
4	Vegetation density around the sampled tree	v_den	unitless	0–1	Measured at tree level: 0—no vegetation in front of the sample, 0.25—some vegetation, 0.5—more vegetation in front of the sample or deeper in an open forest, 0.75—more vegetation and deeper in the forest, 1—more than 5 m in the forest
5	Orientation of the lichen on the tree, with respect to the north	a_north	degrees	0–180	Measured at sample level: 0 = N, 45 = NW = NE, 90 = W = E, 135 = SW = SE, 180 = S
6	Orientation of the lichen on the tree, with respect to the OR direction	a_ref	degrees	0–180	Measured at sample level: 0—in the direction of the OR to 180—180° from the direction of the OR

OR—oil refinery; * raw data for calculating wind direction and speed were obtained from Meteorological and Hydrological Service (DHMZ, [28]) monitoring station Slavonski Brod-1 (2010–2015).
