# Supplementary material for: Any Way the Wind Blows Does Really Matter in Lichen Response to Air Pollution from an Oil Refinery
Source: Toxics. 2025 Feb 25;13(3):160. doi: 10.3390/toxics13030160 (PMC11945780; doi:10.3390/toxics13030160)
Supplement: Supplementary file 1 [file toxics-13-00160-s001.zip › toxics-3490688-supplementary.pdf]

supplementary materials

Any way the wind blows does really matter in lichen response to air pollution from an oil refinery

Maja Maslać Mikulec[1, 2*], Saša Likić[1,3], Oleg Antonić[4,2], Mirta Tkalec[1*]

[1] Department of Biology, Faculty of Science, University of Zagreb, 10 000 Zagreb, Croatia; saslikic@yahoo.com (S.L.)
[2] Geonatura Ltd, 10 000 Zagreb, Croatia; oantonic@geonatura.hr (O.A.)
[3] Biosistemi Grupa Ltd., 10 000 Zagreb, Croatia
[4] Department of Biology, Josip Juraj Strossmayer University of Osijek, 31 000 Osijek, Croatia
* Correspondence: maja.maslachak@gmail.com (MMM); mtkalec@biol.pmf.hr (MT)

Table S1. Bioaccumulation classes for native lichens used in this study (adapted from Cecconi et al. [30]).

Bioaccumulation class	B ratio	Colour code (HTML)
(A) Absence of bioaccumulation	≤ 1.0	#00FF00 (green)
(L) Low bioaccumulation	(1.0, 2.1]	#008000 (dark green)
(M) Moderate bioaccumulation	(2.1, 3.4]	#FFFF00 (yellow)
(H) High bioaccumulation	(3.4, 4.9]	#FF3030 (light red)
(S) Severe bioaccumulation	> 4.9	#FF0000 (red)

Table S2. Correlations of measured variables of lichen vitality with the first four principal components (factor loadings; bold values denote close correlations).

Source variable	F1	F2	F3	F4
F_v/F_m	0.146234	0.039311	-0.045212	**0.955048**
NPQ	-0.038858	**0.990609**	0.095522	-0.013439
Q*p*	0.001545	0.107209	**0.936257**	-0.046996
RFd	-0.090071	**0.989902**	0.038800	0.057282
Total chlorophyll	**0.931957**	-0.028932	-0.161093	0.103281
Phaeophytinization quotient	**0.702255**	0.016626	0.283879	0.222938
Total carotenoids	**0.837803**	-0.054406	-0.218257	-0.179146
Chlorophyll *a*/ chlorophyll *b*	**0.783608**	-0.145376	0.214141	0.230068

F_v/F_m, maximum quantum yield of PSII; NPQ, nonphotochemical quenching; Q*p*, coefficient of photochemical quenching; RFd, fluorescence decrease ratio; total chlorophyll content (mg g^{-1} dw); phaeophytinization quotient; total carotenoid content (mg g^{-1}mg g^{-1} dw); chlorophyll *a* and chlorophyll *b* ratio; F1, F2, F3 and F4, four factors resulting from principal component analysis (PCA).

Table S3. Review-based background element concentration values (BEC; μg g^{-1} dw) for the epiphytic lichen species *Flavoparmelia caperata* [30]. Presented only for selected metals used in this study.

Metal	Ni	Zn	Cd	Pb
BEC	1.27	35.3	0.18	2.37

Table S4. Measured values of dependant variables in sampled native lichens per plot (**mean ± standard deviation**): chlorophyll fluorescence (F_v/F_m - maximum photochemical quantum efficiency of photosystem II, NPQ - nonphotochemical quenching, Q*p* - coefficient of photochemical quenching, RFd- fluorescence decrease ratio); pigments variables in mg g^{-1} dw (Chl *a* - chlorophyll *a*, Chl *b* - chlorophyll *b*, TChl – total chlorophyll, TCar – total carotenoids) and no measuring unit (PQ*a* - phaeophytinization quotient); non-metal (S – sulphur, N – nitrogen) content in mg g^{-1}; metal (Ni – nickel, Zn – zinc, Cd – cadmium, Pb – lead) content in μg g^{-1} dw. Plots are lined up from the closest to the farthest from the refinery. On the plots closest to the refinery (1 and 2) no lichens were found.

Plot	F_v/F_m	Q*p*	NPQ	RFd	Chl *a*	Chl *b*	TChl	PQ*a*
3	0.71±0.02	0.28±0.06	0.82±0.33	0.76±0.32	1.09±0.33	0.43±0.09	1.52±0.41	0.84±0.07
4	0.67±0.03	0.26±0.06	0.92±0.26	0.84±0.25	0.62±0.16	0.29±0.06	0.9±0.23	0.73±0.04
5	0.69±0.05	0.19±0.03	0.61±0.24	0.52±0.24	1.74±0.47	0.66±0.15	2.4±0.62	0.75±0.12
6	0.74±0.01	0.22±0.03	0.71±0.29	0.64±0.27	2.41±0.72	0.86±0.25	3.27±0.97	0.94±0.05
7	0.68±0.03	0.26±0.05	0.83±0.36	0.7±0.32	1.16±0.31	0.44±0.09	1.6±0.39	0.84±0.05
8	0.73±0.01	0.27±0.06	0.64±0.14	0.6±0.14	1.1±0.13	0.46±0.06	1.56±0.18	0.79±0.08
9	0.7±0.03	0.27±0.06	0.71±0.27	0.61±0.23	1.56±0.45	0.55±0.12	2.11±0.57	0.97±0.1
10	0.67±0.06	0.23±0.08	0.87±0.37	0.8±0.32	0.78±0.16	0.34±0.04	1.12±0.2	0.77±0.07
11	0.7±0.03	0.24±0.05	0.91±0.21	0.82±0.18	1.64±0.44	0.59±0.16	2.23±0.59	0.91±0.07
12	0.72±0.02	0.2±0.04	0.85±0.24	0.76±0.21	0.88±0.37	0.41±0.16	1.29±0.44	0.83±0.08
13	0.65±0.04	0.22±0.04	0.78±0.18	0.72±0.18	0.63±0.2	0.3±0.07	0.93±0.26	0.8±0.03
14	0.73±0.01	0.22±0.05	0.71±0.23	0.65±0.24	1.1±0.42	0.42±0.13	1.52±0.54	0.84±0.1
15	0.73±0.02	0.21±0.05	0.66±0.15	0.63±0.13	1.06±0.39	0.42±0.13	1.48±0.52	0.76±0.08
16	0.71±0.05	0.26±0.05	0.84±0.25	0.76±0.25	1.13±0.2	0.43±0.06	1.56±0.25	0.85±0.05
17	0.72±0.01	0.28±0.03	0.63±0.16	0.58±0.13	1.19±0.29	0.45±0.09	1.64±0.38	0.82±0.08
**Plot**	**Chl *a*/ Chl *b***	**TCar**	**N**	**S**	**Ni**	**Zn**	**Cd**	**Pb**
3	2.47±0.37	0.4±0.12	14.62±3.78	1.66±0.32	6.12±2.23	50.6±9.05	0.12±0.04	5.15±2.79
4	2.15±0.18	0.31±0.06	8.44±2.31	1.35±0.13	3.61±0.59	34.43±6.67	0.4±0.1	5.34±0.73
5	2.6±0.21	0.62±0.05	21.04±5.88	2.01±0.27	4.82±0.84	80.5±23.56	0.17±0.03	7.34±2.05
6	2.81±0.21	0.49±0.11	20.92±3.36	1.8±0.24	6.28±3.22	55.6±12.34	0.15±0.04	11.13±4.06
7	2.64±0.27	0.36±0.08	15.81±1.06	1.53±0.05	3.54±2.23	38.37±6.15	0.5±0.24	5.48±1.51
8	2.41±0.23	0.41±0.05	13.97±3.24	1.65±0.3	2.54±0.46	30.83±9.29	0.5±0.62	2.61±0.28
9	2.81±0.27	0.44±0.06	17.98±4.47	1.64±0.12	2.4±0.78	40±12.08	0.12±0.02	3.41±1.3
10	2.32±0.28	0.3±0.03	9.5±0.54	1.31±0.05	1.97±0.26	25.47±3.84	0.25±0.03	8.62±0.83
11	2.79±0.14	0.49±0.12	17.6±3.03	1.63±0.11	9.21±5.58	30.1±12.47	0.28±0.15	5.05±1.13
12	2.29±0.73	0.29±0.09	10.79±2.28	1.39±0.07	3.8±0.59	27.37±4.1	0.43±0.1	5.91±2.41
13	2.09±0.31	0.28±0.07	9.19±3.87	1.34±0.14	3.63±0.96	19.37±4.02	0.32±0.09	5.68±0.93
14	2.52±0.36	0.35±0.08	14.67±1.32	1.52±0.11	3.06±1.15	43±14.42	0.22±0.01	8.62±1.2
15	2.49±0.22	0.38±0.1	17.31±1.82	1.56±0.12	3.2±1.78	53.3±13.11	0.23±0.09	4.04±0.51
16	2.6±0.27	0.42±0.08	15.97±4.76	1.57±0.3	2.81±0.45	48.4±13.91	0.11±0.02	3.65±0.71
17	2.64±0.21	0.42±0.07	13.86±3.39	1.63±0.44	2.85±1.02	25.5±6.9	0.18±0.06	5.79±2.13

Table S5. Measured values of dependant variables in sampled native lichens per plot (**median; range**); variable names explained in previous table (Table S4). Plots are lined up from the closest to the farthest from the refinery. On the plots closest to the refinery (1 and 2) where no lichens were found.

Plot	F_v/F_m	Q*p*	NPQ	RFd	Chl *a*	Chl *b*	TChl	PQ*a*
3	0.71; 0.66-0.74	0.27; 0.19-0.36	0.76; 0.44-1.69	0.74; 0.37-1.58	1.05; 0.5-1.53	0.46; 0.25-0.52	1.52; 0.74-2.05	0.81; 0.77-0.97
4	0.67; 0.62-0.72	0.25; 0.18-0.35	0.84; 0.58-1.36	0.77; 0.54-1.26	0.63; 0.41-0.85	0.29; 0.2-0.36	0.93; 0.61-1.2	0.73; 0.68-0.8
5	0.69; 0.6-0.74	0.2; 0.15-0.26	0.54; 0.38-1.19	0.45; 0.29-1.1	1.61; 1.23-2.35	0.65; 0.51-0.87	2.27; 1.74-3.22	0.7; 0.63-0.92
6	0.74; 0.72-0.76	0.22; 0.17-0.26	0.6; 0.45-1.43	0.53; 0.39-1.34	2.16; 1.67-3.58	0.76; 0.67-1.26	2.92; 2.33-4.77	0.95; 0.84-0.99
7	0.7; 0.61-0.71	0.25; 0.19-0.34	0.88; 0.41-1.57	0.71; 0.28-1.36	1.12; 0.8-1.82	0.45; 0.32-0.63	1.59; 1.12-2.46	0.84; 0.77-0.94
8	0.73; 0.71-0.75	0.26; 0.21-0.38	0.6; 0.52-0.95	0.57; 0.47-0.92	1.11; 0.88-1.3	0.45; 0.35-0.56	1.57; 1.31-1.78	0.8; 0.65-0.9
9	0.7; 0.66-0.74	0.28; 0.19-0.4	0.66; 0.38-1.1	0.57; 0.32-0.92	1.48; 0.71-2.26	0.52; 0.3-0.72	2.01; 1.02-2.99	0.92; 0.86-1.11
10	0.68; 0.58-0.74	0.23; 0.11-0.35	0.92; 0.38-1.47	0.87; 0.35-1.32	0.88; 0.49-0.97	0.34; 0.27-0.42	1.22; 0.76-1.4	0.77; 0.65-0.87
11	0.7; 0.65-0.73	0.26; 0.14-0.29	0.91; 0.61-1.34	0.83; 0.57-1.19	1.45; 1.16-2.38	0.55; 0.43-0.89	2; 1.59-3.27	0.93; 0.77-0.98
12	0.71; 0.69-0.75	0.21; 0.12-0.26	0.87; 0.55-1.22	0.78; 0.45-1.09	0.87; 0.4-1.62	0.36; 0.24-0.76	1.23; 0.63-2.22	0.83; 0.71-0.94
13	0.68; 0.59-0.69	0.23; 0.16-0.28	0.79; 0.54-1.09	0.73; 0.48-0.99	0.63; 0.3-0.97	0.33; 0.16-0.38	0.95; 0.46-1.35	0.79; 0.77-0.86
14	0.73; 0.71-0.74	0.2; 0.16-0.32	0.63; 0.52-1.18	0.56; 0.47-1.15	1.17; 0.48-1.73	0.42; 0.26-0.62	1.6; 0.75-2.34	0.87; 0.61-0.95
15	0.73; 0.67-0.75	0.2; 0.14-0.27	0.71; 0.48-0.86	0.67; 0.45-0.82	1.19; 0.54-1.49	0.45; 0.23-0.56	1.65; 0.76-2.05	0.78; 0.62-0.86
16	0.73; 0.62-0.76	0.26; 0.18-0.34	0.81; 0.52-1.36	0.8; 0.47-1.29	1.15; 0.64-1.34	0.45; 0.3-0.51	1.62; 0.94-1.84	0.86; 0.72-0.93
17	0.71; 0.7-0.75	0.29; 0.22-0.32	0.64; 0.4-0.92	0.6; 0.4-0.76	1.21; 0.84-1.63	0.46; 0.33-0.61	1.68; 1.18-2.19	0.84; 0.69-0.91
**Plot**	**Chl *a*/ Chl *b***	**TCar**	**N**	**S**	**Ni**	**Zn**	**Cd**	**Pb**
3	2.4; 2.02-2.93	0.43; 0.13-0.5	15.8; 8.74-18.82	1.64; 1.2-2.08	6.23; 3.84-8.29	50.9; 41.4-59.5	0.11; 0.09-0.16	4.63; 2.66-8.17
4	2.12; 1.91-2.38	0.33; 0.21-0.37	7.78; 6.59-11.62	1.33; 1.21-1.51	3.86; 2.94-4.04	32.7; 28.8-41.8	0.39; 0.31-0.5	4.97; 4.88-6.18
5	2.57; 2.4-2.98	0.61; 0.57-0.69	22.26; 14.98-28.81	2.08; 1.62-2.27	4.66; 4.07-5.72	93.5; 53.3-94.7	0.16; 0.15-0.2	7.99; 5.04-8.99
6	2.82; 2.5-3.08	0.54; 0.31-0.62	21.18; 16.97-24.34	1.8; 1.52-2.08	4.5; 4.35-10	53.9; 44.2-68.7	0.17; 0.11-0.18	9.72; 7.96-15.7
7	2.67; 2.22-2.94	0.36; 0.24-0.49	16.26; 14.6-16.57	1.52; 1.48-1.58	2.66; 1.88-6.08	35.7; 34-45.4	0.62; 0.22-0.65	5.92; 3.8-6.73
8	2.4; 2.07-2.76	0.42; 0.33-0.47	12.56; 11.63-19.89	1.62; 1.33-2.07	2.75; 2.02-2.86	32.3; 20.9-39.3	0.15; 0.13-1.22	2.57; 2.35-2.9
9	2.84; 2.35-3.19	0.44; 0.34-0.53	16.31; 14.73-24.56	1.63; 1.52-1.76	2.07; 1.85-3.29	44.6; 26.3-49.1	0.12; 0.11-0.14	3.47; 2.08-4.68
10	2.36; 1.85-2.63	0.28; 0.26-0.34	9.47; 8.98-10.07	1.34; 1.25-1.34	1.92; 1.74-2.26	27; 21.1-28.3	0.26; 0.21-0.27	8.39; 7.93-9.54
11	2.79; 2.5-2.96	0.49; 0.33-0.66	19.25; 14.1-19.44	1.65; 1.51-1.72	10.4; 3.13-14.1	25.6; 20.5-44.2	0.2; 0.18-0.45	4.7; 4.13-6.31
12	2.58; 0.61-2.93	0.29; 0.16-0.49	9.6; 9.35-13.41	1.42; 1.31-1.43	3.61; 3.33-4.46	27.3; 23.3-31.5	0.41; 0.35-0.54	5.09; 4.02-8.62
13	2.05; 1.56-2.52	0.31; 0.18-0.37	8.01; 5.96-14.81	1.3; 1.21-1.53	3.22; 2.94-4.72	20.5; 14.9-22.7	0.3; 0.25-0.42	5.8; 4.7-6.54
14	2.6; 1.75-2.86	0.32; 0.25-0.46	13.97; 13.85-16.2	1.52; 1.42-1.63	2.56; 2.25-4.37	48.7; 26.6-53.7	0.22; 0.21-0.23	9; 7.28-9.59
15	2.57; 2.1-2.68	0.37; 0.23-0.53	17.86; 15.28-18.8	1.58; 1.44-1.67	2.34; 2.01-5.25	47.6; 44-68.3	0.2; 0.17-0.33	4.16; 3.48-4.47
16	2.66; 2.1-2.91	0.4; 0.3-0.58	15.18; 10.67-23.63	1.52; 1.29-2.06	2.87; 2.34-3.23	55.2; 32.4-57.6	0.1; 0.09-0.13	3.65; 2.94-4.35
17	2.57; 2.39-3.01	0.45; 0.3-0.5	13.96; 10.08-17.41	1.47; 1.31-2.27	2.49; 2.05-4	23; 20.2-33.3	0.16; 0.13-0.24	6.18; 3.49-7.69

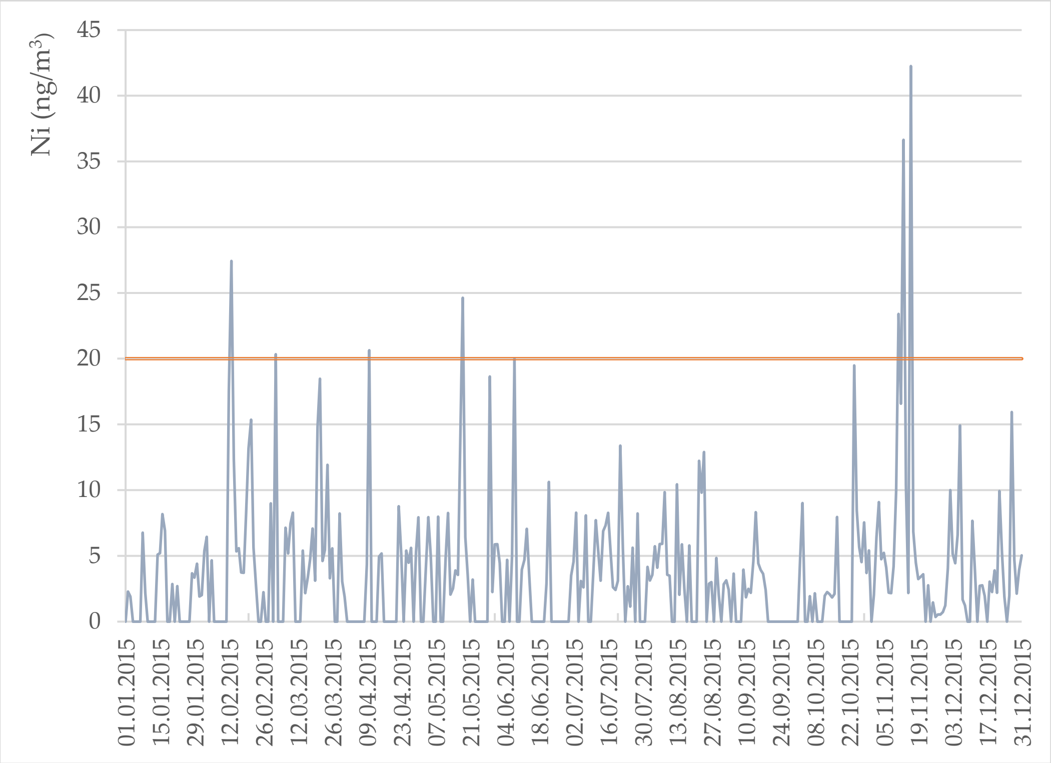

Figure S1. Temporal dynamics of average nickel (Ni) concentrations (ng mg^{-3}) in the PM_{10} fraction at the Slavonski Brod-1 monitoring station in 2015. when these measurements started. Red line represents limit value for the yearly average according to European Union legislation. Data for creating this graph were downloaded from Croatian Agency for Environment and Nature. Air Quality Database [28].

References

28. Croatian Agency for Environment and Nature. Air Quality Database http://iszz.azo.hr/iskzl/index.html (accessed Sep 15, **2016**).

30. Cecconi, E.; Fortuna, L.; Benesperi, R.; Bianchi, E.; Brunialti, G.; Contardo, T.; Nuzzo, L. Di; Frati, L.; Monaci, F.; Munzi, S.; et al. New Interpretative Scales for Lichen Bioaccumulation Data: The Italian Proposal. *Atmosphere (Basel).*, **2019**, *10* (3). https://doi.org/10.3390/atmos10030136.
